# Distinct motivational factors shape news-seeking in young adults

**DOI:** 10.1038/s41598-026-39354-1

**Published:** 2026-06-30

**Authors:** Ellen M. O’Donoghue, Kathrin C. J. Eschmann, Hikaru Tsujimura, Bonni Crawford, David Caswell, Suzanne Oosterwijk, Matthias J. Gruber

**Affiliations:** 1 Cardiff University Brain Research Imaging Centre (CUBRIC), School of Psychology, Cardiff University, Maindy Rd., Cardiff, CF24 4HQ UK; 2 BBC News Labs, New Broadcasting House, London, UK; 3 Social Psychology Department, University of Amsterdam, Amsterdam, The Netherlands

**Keywords:** Human behaviour, Psychology

## Abstract

Researchers and news providers have increasingly aimed to identify the distinct motivational factors that promote news-seeking behaviour. However, existing theoretical approaches are rarely data-driven and existing media strategies have not been empirically tested. Here, we created 24 questionnaire items capturing a range of motivations that might support active news-seeking among an undergraduate sample (Study 1). Through factor analysis, we identified three distinct motivational factors: ‘Informational Updating’, ‘Understanding & Sense-Making’, and ‘Affect Regulation’. Among these factors, ‘Informational Updating’ was most strongly predictive of young adults’ news-seeking frequency. Furthermore, this relationship could not be explained by individuals’ general tendencies to express curiosity (i.e., trait curiosity). In a pre-registered follow-up study (Study 2), we next asked whether the same factors that promote general news-seeking would also promote news-seeking surrounding a specific world event: the February 2022 invasion of Ukraine. Although all three motivational factors individually predicted news-seeking frequency surrounding the invasion, only the influence of ‘Understanding & Sense-Making’ remained significant while controlling for the remaining factors. Our data suggest that the desire to stay informed may be the strongest driver of general news-seeking tendencies, but that other motivational factors can take precedence for specific, major news topics.

## Introduction

Never before in human history have there been so many opportunities to stay informed about local and global current affairs. To efficiently navigate the abundance of headlines that are encountered in any given day, consumers must selectively engage with the content that they perceive as most worthwhile while avoiding informational overload^1^ and news fatigue^2^. Identifying the distinct motivational factors that promote news consumption thus has the potential to aid both news providers and consumers in promoting healthy news hygiene. Of particular interest are the factors that underlie consumers’ general tendencies towards news consumption, over and above the factors that might drive individuals to follow specific news stories.

Longstanding bodies of both theory and practice have made substantial headway in characterising why people consume news. For example, Uses & Gratifications Theory proposes that individuals actively select media that fulfil specific needs—including drives to stay informed, reduce stress, and maintain social connections^3–5^, among others. Additionally, several news providers have developed frameworks that tailor and classify content according to consumers’ perceived motivations. For example, the British Broadcasting Corporation (BBC) identified six user needs that might promote news-seeking: “update me”, “keep me on trend”, “inspire me”, “divert me”, “educate me”, and “give me perspective”^6^. To our knowledge, neither this nor similar practitioner frameworks have been empirically tested outside of their operational use.

Importantly, and in the context of news-seeking, such frameworks are typically applied in a top-down manner. Researchers and practitioners begin by specifying specific motivations that are expected to drive news consumption, and then examine the extent to which participants’ actual behaviours map onto them. By contrast, the present work adopts a bottom-up, data-driven approach. Rather than testing specific, established theoretical frameworks, we aim to empirically derive the motivational factors that emerge from participants’ self-reported news-seeking motivations. This approach provides an opportunity to test the extent to which theoretically proposed motivations—such as informational updating, affect regulation, or social connectedness, among others—are reflected in the latent psychological structure that underpins participants’ responses and, as such, it serves as a complementary approach toward understanding which factors distinctly motivate news-seeking.

Here, we focus specifically on active news-seeking, that is, deliberate, goal-directed engagement with news content, rather than passive or incidental exposure. Existing theoretical perspectives from journalism and experimental psychology have suggested several motivations that could plausibly shape such behaviour, including *informational updating*, *understanding and sense-making*, *affect regulation*, and *social connectedness* among others.

First, *informational updating*—a term that we use to encompass factors such as surveillance motivations^7,8^, the desire to stay informed^9^, and the “need to know”^10^—is generally believed to be one of the key motivational factors underlying active news-seeking behaviour^10–13^. In many cases, informational updating is driven by the search for instrumental utility (i.e., for information that can be used to adaptively modify behaviour;^14^. However, research on information seeking has demonstrated that non-instrumental information—which is not immediately useful for behavioural adaptation—also has reinforcing value^15^ and might likewise contribute to informational updating tendencies across multiple domains^16^.

Second, and beyond this drive to remain generally up-to-date, news consumers might be motivated to resolve specific information gaps by incorporating newly obtained information into their existing mental schemas (see^17^, for related discussion surrounding this distinction). These schemas then help readers to better understand and act within the world^18^. This process of *understanding & sense-making* appears to be largely automatic, though it can also drive controlled information-seeking: when someone encounters a salient information gap, they may be strongly motivated to resolve that gap^19–21^. However, research on sense-making also suggests that readers should be selective in which content they consume^1,19^. Under the assumption that sense-making requires cognitive effort, it is more economical for the brain to maintain its existing models of the world than to seek alternative models. Therefore, the precise circumstances in which motivations for understanding & sense-making might promote news-seeking remain unclear.

Importantly, the above distinction between shallow awareness and deeper cognitive engagement is also central to the Cognitive Mediation Model (CMM;^7^, which posits that meaningful learning from news depends on two key processes: attention to news content and elaboration on that content. From this perspective, informational updating may reflect relatively superficial monitoring, whereas understanding & sense-making may reflect deeper forms of cognitive processing (i.e., elaborative integration).

Third, news articles can be useful for *affect regulation*. Positively-valenced articles can enhance positive affect^22^, while long-term exposure to negatively-valenced news can increase stress and anxiety^23^. These negative effects can lead to news avoidance, which in turn alleviates negative mood and thus serves as a negative reinforcer^24^. Importantly, affect regulation can be an active process. Individuals may deliberately engage a variety of strategies that allow them to enhance positive affect and/or to alleviate negative affect^25,26^.

Finally, news can serve a clear social function^10^. Regular news consumption allows readers to keep up-to-date on what is happening in their communities and to be informed participants in news-related conversations. Thus, news-seeking can reduce feelings of social isolation while increasing both social and societal *connectedness*^27^.

In addition to these motivational factors, a separate, nascent line of psychological research has investigated the impact of curiosity on information seeking. Broadly defined as a desire to seek information in absence of external rewards^28–30^, curiosity has been shown to promote information seeking across various experimental paradigms^31–33^. Importantly, Eschmann, Pereira et al.^34^ found that individual differences in trait curiosity predicted real-world news seeking during the COVID-19 pandemic, suggesting that curiosity might likewise predict general news-seeking behaviour.

Curiosity is closely connected to the four motivational factors discussed above. Just as motivations for informational updating have been proposed to drive the search for non-instrumental information, so too has curiosity^35^. Curiosity can be elicited by salient information gaps^32^, implicating it in the drive for sense-making, and it can drive individuals to seek out negative content^36,37^. Additionally, social curiosity and a desire for gossip may feed into social motivations for news consumption^38^. Given that people exhibit stable individual differences in their propensities to experience these facets of curiosity^34,39^, it remains unclear whether the motivational factors underlying news consumption are independent of trait curiosity, or whether they might instead be manifestations of it.

Relatedly, and despite the many theoretical and practical advancements described above, it is not yet clear which motivational factors make distinct contributions to news-seeking behaviour. In the present study, we took a bottom-up, data-driven approach towards identifying the motivational factors that promote news-seeking. We considered both general news-seeking (Study 1) and news-seeking surrounding a specific world event: the Russia-Ukraine War (Study 2).

## Study 1

In Study 1, we created 24 items to probe the motivational factors underlying general news-seeking (Table 1). The first 15 items listed in Table 1 were based on the BBC’s existing news frameworks^3^ and intended to reflect the four potential motivational factors discussed above: informational updating^7–10,12–16^, understanding & sense-making^1,14,17–21^], affect regulation^22–26^, and connectedness^10,27^. In addition, we included items related to a range of theoretical ideas and recent empirical findings around information seeking drawn from psychology and neuroscience, including insight^40^, impulsivity^41^, self-efficacy^42^, agency^43^, self-relevance^8^, moral duty^13^, and news avoidance^44^. Our aim was to capture a broad range of possible motivations for news-seeking, with particular emphasis on the factors that are currently emphasized by news providers such as the BBC.

**Table 1:** The 24 items used in study 1.

Questionnaire item	Classification
I read articles that…	
Keep me up to date on a current topic.	Informational updating
Tell me something specific that I want to know.	Informational updating
Give me the latest information.	Informational updating
Give me a different perspective on the world.	Understanding & sense-making
Help me to understand the world better,	Understanding & sense-making
Help me to formulate an opinion.	Understanding & sense-making
Confirm my opinion,	Understanding & sense-making
Are thought-provoking.	Understanding & sense-making
Lift my mood.	Affect regulation
Engage me emotionally.	Affect regulation
Inspire me.	Affect regulation
Relax me.	Affect regulation
Make me feel connected to other people.	Connectedness
Help me to share the experience of other people.	Connectedness
Give me something to talk about with other people.	Connectedness
Give me insight.	Insight
On impulse (without thinking).	Impulsivity
Can help me to adjust my behaviour in our changing world.	Self-efficacy
Can help me to make better decisions in our changing world.	Agency
Are relevant to my own personal situation.	Personal relevance
Because I feel it is my moral duty to inform myself.	Moral duty
I avoid articles that…	
Challenge my opinion.	Avoidance
Are complicated.	Avoidance
Could make me feel distressed.	Avoidance

Based on participants’ responses to these items, we conducted an exploratory factor analysis to identify the distinct motivational factors underlying news-seeking. We next asked whether these factors predicted news-seeking frequency, as well as whether their influence extended beyond the influence of trait curiosity.

## Method

### Participants

We recruited 312 participants between the ages of 18 and 25 from Cardiff University’s subject pool. Our sample size was determined in accordance with the recommendations for factor analysis provided by^45^. Participants provided their informed consent and received course credit in exchange for participation. All procedures were approved by Cardiff University’s School of Psychology Research Ethics Committee and performed in accordance with the relevant guidelines and regulations. We excluded one participant who reported scores of 0 on every item, resulting in a final *N* = 311 (mean age = 19.27 years, *SD* = 1.49; 265 female, 29 male, 17 nonbinary/”other”). Participants completed the study online via Qualtrics using either their phone or personal computer.

### Materials and methods

We considered 24 questionnaire items (Table 1). The items were presented to participants in random order and participants were asked to respond to each item using sliding scales that ranged from 0 (“not at all applicable”) to 100 (“strongly applicable”). As a measure of trait curiosity, participants also completed the 24-item revised Five-Dimensional Curiosity Scale (5DCR;^39^, which consists of six subscales corresponding to joyous exploration, deprivation sensitivity, thrill seeking, stress tolerance, overt social curiosity, and covert social curiosity. In our sample, each subscale had strong internal consistency (mean Cronbach’s α = 0.83; minimum = 0.77, maximum = 0.86). Finally, participants reported their own news consumption frequency (“how often do you consume news?”) on a 4-point Likert-type scale (1 = “never”; 2 = “rarely”; 3 = “weekly”, 4 = “daily”). Mean consumption frequency was relatively high (mean = 3.13, *SD* = 0.83), and the measure had slight negative skew (= -0.38).

### Exploratory factor analysis

To determine the factor structure of our 24 items, we conducted an exploratory factor analysis using the ‘psych’ package for RStudio^46^. We began by assessing whether our data were suitable for factor analysis using the Kaiser-Meyer-Olkin (KMO) test. In line with the guideline that KMO factors > 0.60 indicate good suitability^47^, we determined that they were (observed KMO = 0.84).

In deciding the number of factors to retain, we used a data-driven approach comprising multiple decision rules: visual scree plot (VSP) analysis, parallel analysis using Principal Axis Factoring (PAF), and Velicer’s Minimum Average Partial (MAP) test^47^. Parallel analysis suggested the retention of six factors, whereas both the VSP analysis and the MAP test suggested the retention of three factors. For parsimony, we thus opted for a three-factor solution.

Finally, we conducted a three-factor analysis using maximum likelihood estimation. Because we expected the resultant factors to be intercorrelated, we applied direct quartimin (‘oblimin’) rotation. We then determined the appropriate factor loadings using the 0.40-0.30-0.20 rule^47^, which specifies that (1) primary factor loadings should exceed 0.40, (2) alternative factor loadings should *not* exceed 0.30, and (3) the difference between primary and alternative factor loadings should be at least 0.20.

## Results

### Three distinct motivational factors underlie news-seeking

**Fig. 1:**
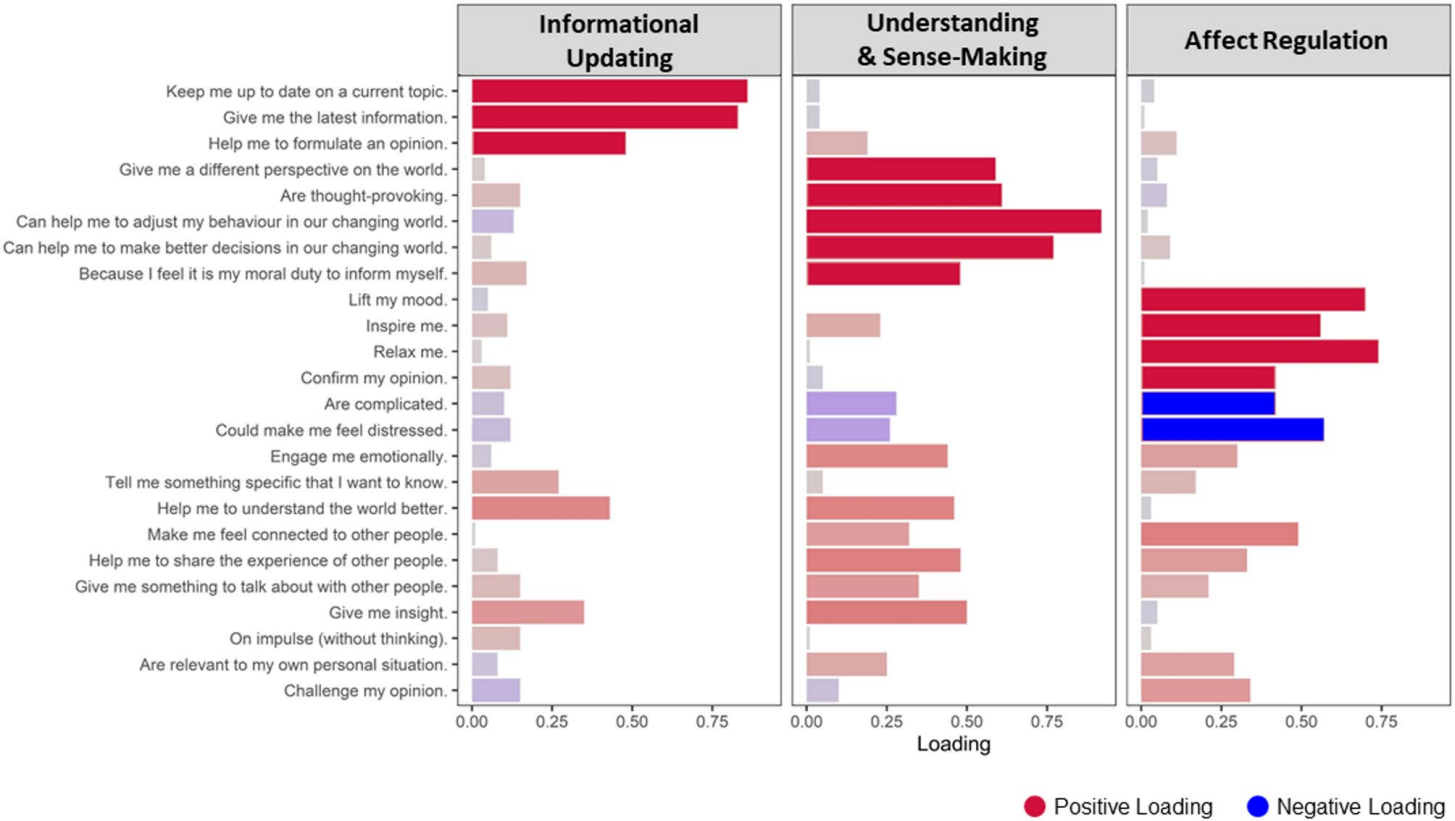
Factors loadings along each of the three identified factors—‘Informational Updating’, ‘Understanding & Sense-Making’, and ‘Affect Regulation’– for all 24 items considered in Study 1. Red bars indicate positive factor loadings; blue bars indicate negative factor loadings. The bolded bars correspond to items that successfully loaded onto one of the three factors according to the 0.40-0.30-0.20 rule^47^.

Based on our exploratory factor analysis, we retained 14 of our original 24 items (Fig. 1). These items loaded onto three factors. The first factor, which we labelled as ‘Informational Updating’, comprised three items related to the desire to stay informed (e.g., “I read articles that keep me up-to-date on a current topic”). The second factor, labelled as ‘Understanding & Sense-Making’, comprised five items related to perspective-seeking as well as behavioural adjustment (e.g., “I read articles that give me a different perspective on the world”; “I read articles that can help me to adjust my behaviour in our changing world”). The third factor, labelled as ‘Affect Regulation’, comprised six items related to both promoting positive affect and preventing negative affect (e.g., “I read articles that lift my mood”; “I avoid articles that could make me feel distressed”), reflecting a broad underlying motivation to manage affective responses (rather than specific tendencies to approach or avoid valenced content).

Perhaps surprisingly, we did not find evidence for ‘Social Connectedness’ as a separate motivational factor; additionally, the questionnaire items that were intended to tap into social connectedness motivations did not load on to any of the three factors that we identified. We suspect that this finding reflects the fact that younger age groups (including our young adult sample) may prefer to fulfil their motivations for social connectedness from alternative outlets, such as social media^58^, rather than from traditional news outlets (which were the focus of the present study). Indeed, given that young people are increasingly less likely to get their news from traditional news platforms^46^, the ability to connect with same-age peers and communities through those platforms is likely to be limited.

To assess the correlations among the three motivational factors (and for all subsequent analyses), we excluded ten participants who were more than three standard deviations beyond the mean of any measure of interest [i.e., motivational factors: ‘Informational Updating’, ‘Understanding & Sense-Making’, ‘Affect Regulation’; 5DCR subscales: joyous exploration, deprivation sensitivity, thrill-seeking, stress tolerance, overt social curiosity, and covert social curiosity^39^. We thus retained 302 participants for further analysis. We then determined participants’ scores for each factor by calculating the means of their responses across all items that loaded onto that factor according to the 0.40-0.30-0.20 rule^47^; Fig. 1).

Using a series of Pearson’s correlations with Bonferroni corrections to adjust for multiple comparisons, we determined that ‘Informational Updating’ was significantly positively correlated with ‘Understanding & Sense-Making’, *r*(300) = 0.45, 95% CI: [0.36, 0.54], adjusted *p* < .001, but significantly negatively correlated with ‘Affect Regulation’, *r*(300) = − 0.15, 95% CI: [-0.25, − 0.03], adjusted *p* = .033. The correlation between ‘Understanding & Sense-Making’ and ‘Affect Regulation’ was not significant, *r*(300) = − 0.02, 95% CI [− 0.13, 0.09], adjusted *p* > 1.00.

The fact that participants who were more motivated to acquire information (i.e., participants with higher scores along ‘Informational Updating’) were also more motivated to make sense of that information (i.e., higher scores along ‘Understanding & Sense-Making’) is consistent with the theoretical perspective that the drive for sense-making can feed into more general tendencies towards information seeking^19^. The negative correlation between ‘Informational Updating’ and ‘Affect Regulation’ also suggests that strong motivations to maintain positive affect (i.e., higher scores along ‘Affect Regulation’) might compete against this general information-seeking tendency^49^.

### ‘Informational updating’ and ‘understanding & sense-making’ individually predict general news consumption frequency, but ‘affect regulation’ does not

**Fig. 2:**
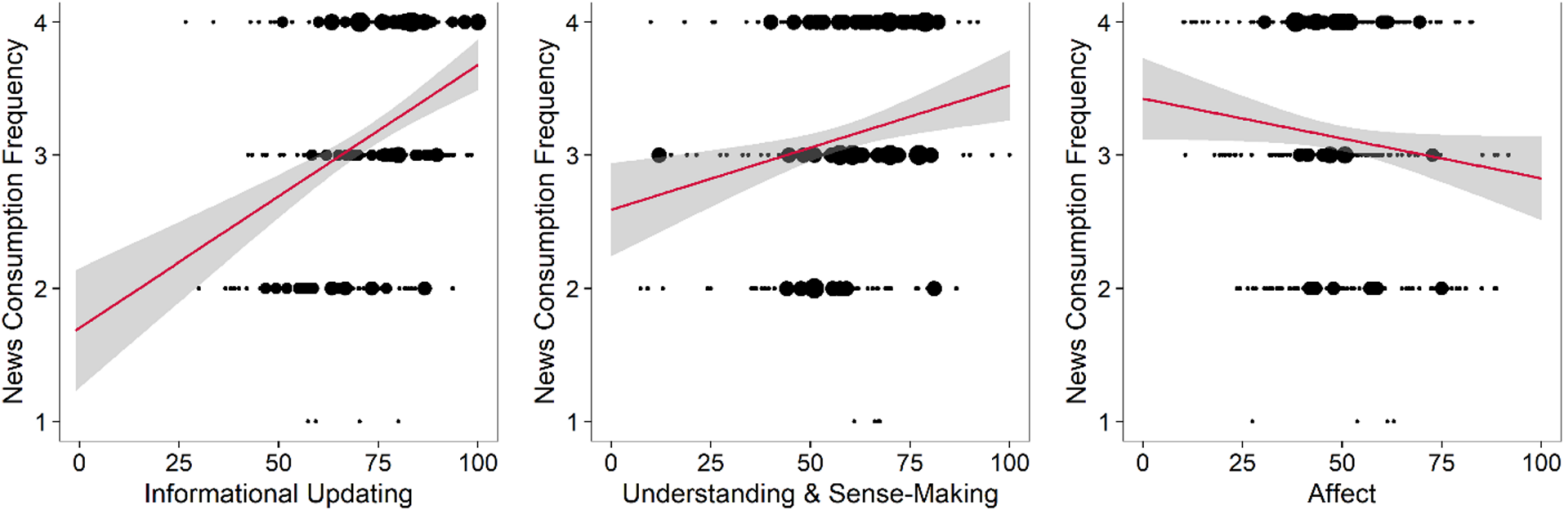
The three identified motivational factors (‘Informational Updating’, ‘Understanding & Sense-Making’, and ‘Affect Regulation’) as individual predictors of news consumption frequency (1 = “never”, 2 = “rarely”, 3 = “weekly”, 4 = “daily”). The points correspond to real data, with the size of the points reflecting the number of observations at each level.

To assess whether the motivational factors identified above individually predicted the frequency of news consumption, we conducted three separate linear regressions (Fig. 2). As above, we used Bonferroni correction to adjust for multiple comparisons. Both ‘Informational Updating’ and ‘Understanding & Sense-Making’ were significant positive predictors of news consumption frequency [‘Informational Updating’: *b* = 0.02, 95% CI [0.01, 0.03], *t*(300) = 6.38, adjusted *p* < .001; ‘Understanding & Sense-Making’: *b* = 0.01, 95% CI [< 0.01, 0.02], *t*(300) = 3.15, adjusted *p* = .005], demonstrating that participants consumed news more often if they were motivated to stay informed and/or to acquire greater understanding. Conversely, ‘Affect Regulation’ was not significantly predictive of general news consumption frequency after Bonferroni correction, *b* = − 0.01, 95% CI [− 0.01, >− 0.01], *t*(300) = − 2.00, adjusted *p* = .140.

### ‘Joyous exploration’ predicts news consumption frequency over and above other curiosity subscales

We additionally sought to replicate the relationship previously found between trait curiosity and news consumption frequency^34^. To do so, we conducted a multiple regression examining each of the six 5DCR subscales—joyous exploration, deprivation sensitivity, thrill seeking, stress tolerance, overt social curiosity, and covert social curiosity^39^—as potential predictors of news consumption frequency. For consistency with further planned analyses (see below), all variables were z-scored prior to analysis. Examination of the Variance Inflation Factors (VIFs) indicated that multicollinearity was not a concern, all VIFs < 1.42.

**Fig. 3:**
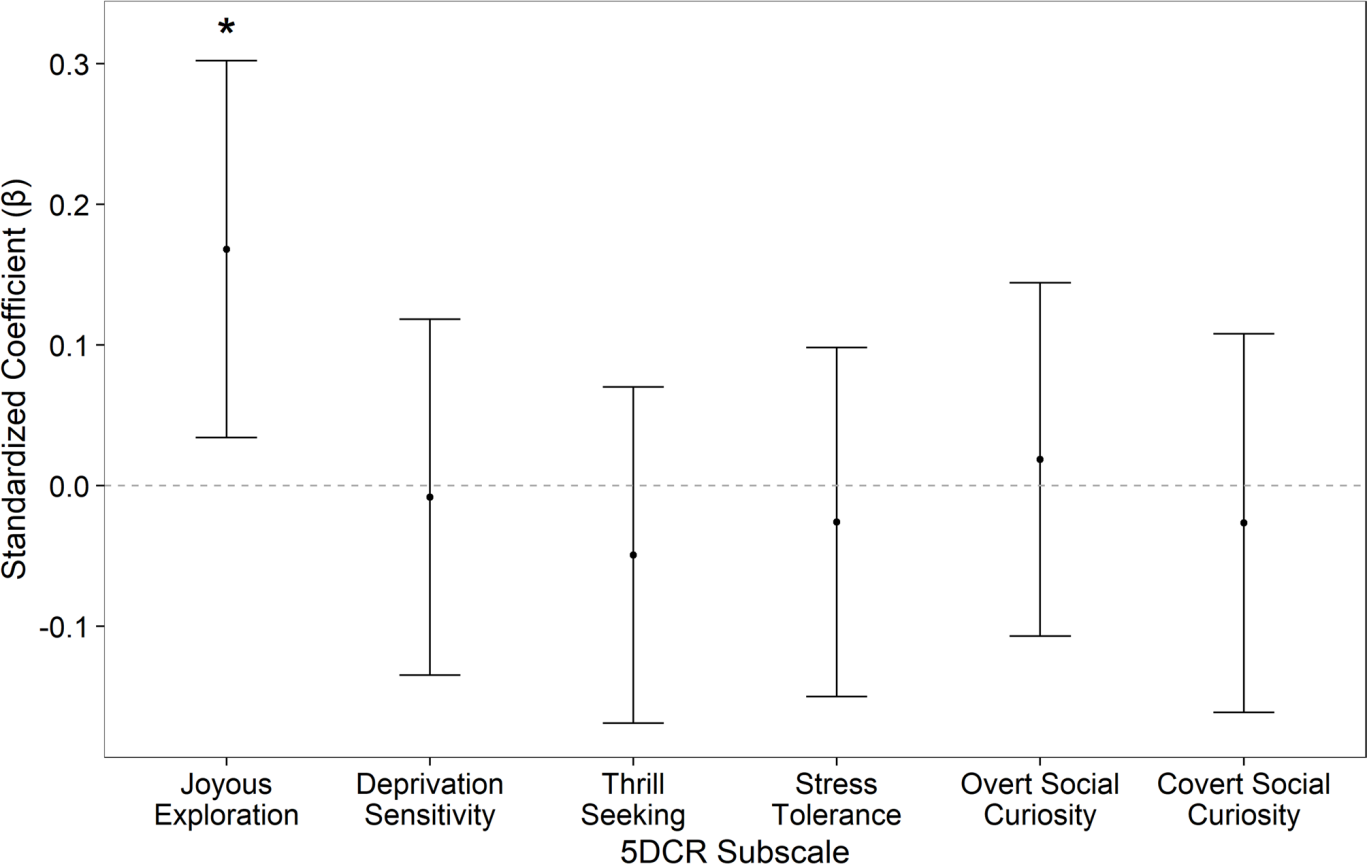
Among the 5DCR subscales, only joyous exploration significantly predicted news consumption. The points depict standardized regression coefficients (βs) for each of the 5DCR subscales^39^ used to predict news consumption frequency: joyous exploration, deprivation sensitivity, thrill-seeking, stress tolerance, overt social curiosity, and covert social curiosity. Error bars reflect 95% CIs and asterisks denote statistical significance (* *p* < .050).

Joyous exploration was a significant, positive predictor of news consumption frequency, β = 0.17, 95% CI [0.03, 0.30], *t*(295) = 2.47, *p* = .014, whereas none of the other subscales significantly predicted news consumption frequency, all *p* values > 0.416 (Fig. 3). Prior research has also suggested that joyous exploration plays a role in real-world news seeking^34^.

### ‘Informational updating’ is the strongest predictor of general news consumption frequency

**Fig. 4:**
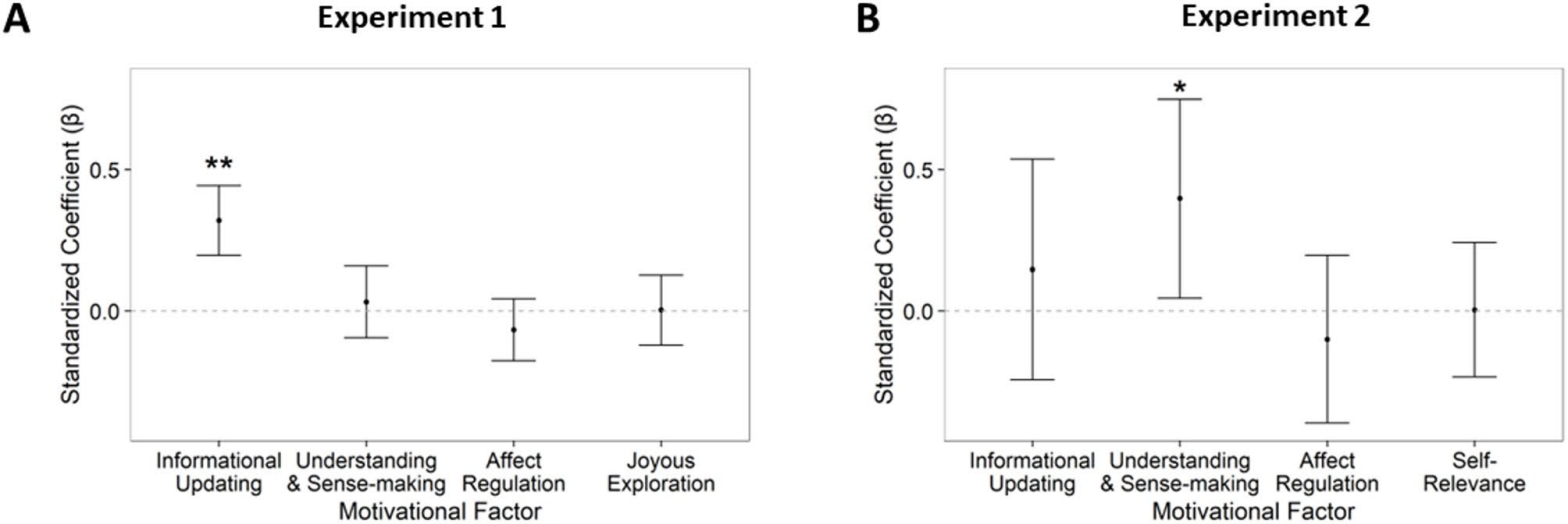
Informational Updating was the strongest predictor of general news consumption in Study 1 (A), while ‘Understanding & Sense-Making’ was the strongest predictor of news consumption surrounding the Russia-Ukraine War in Study 2 (B). The points depict standardized regression coefficients (βs) for each of the motivational factors used to predict news consumption frequency. Error bars reflect 95% CIs and asterisks denote statistical significance (* *p* < .050; *** *p* < .001).

Finally, to assess whether the identified motivational factors predict news consumption frequency over and above the effects of trait curiosity, we conducted a multiple regression using all three motivational factors (‘Informational Updating’, ‘Understanding & Sense-Making’, ‘Affect Regulation’) and joyous exploration. Multicollinearity was not a concern, all VIFs < 1.41, and all variables were z-scored prior to analysis. In this model, only ‘Informational Updating’ remained a significant predictor of news consumption frequency, β = 0.32, 95% CI [0.20, 0.44], *t*(297) = 5.11, *p* < .001, all other *p* values > 0.237 (Fig. 4A). Our data thus support that ‘Informational Updating’ was the strongest predictor of participants’ general news consumption: participants who were more motivated to stay up-to-date reported consuming news more frequently.

## Study 2

In Study 1, we identified three motivational factors that promote news consumption: ‘Informational Updating’, ‘Understanding and Sense-Making’, and ‘Affect Regulation’. Although all three factors individually predicted news consumption frequency among our undergraduate sample, we found that ‘Informational Updating’ was the strongest predictor when the remaining factors were controlled. Additionally, the influence of ‘Informational Updating’ extended beyond the influence of trait curiosity.

Next, in Study 2, we asked whether the motivational factors identified in Study 1 might also predict news consumption surrounding specific world events. Specifically, we examined participants’ consumption of news surrounding the 2022 escalation of the Russia-Ukraine War.

In the context of news-seeking, the Russia-Ukraine War is notable (1) because of its global impact, and (2) because of the high degree of uncertainty that surrounded the full-scale invasion of Ukraine in February 2022. Prior research surrounding the COVID-19 pandemic—an event similarly marked by its global impact and uncertainty—highlighted ‘Self-Relevance’ as one of the key motivational factors underlying news consumption^13^. We thus opted to examine the possible influence of ‘Self-Relevance’ alongside the three motivational factors identified in Study 1.

Our analysis plan was preregistered (see https://osf.io/pab42/). In this paper, we have included only the elements of our preregistration that were intended as a direct follow-up to our findings from Study 1.

## Method

### Participants

We recruited participants from a pre-existing database at the Cardiff University Brain Research Imaging Centre. In accordance with our preregistered analysis plan, we opted to use this database because we were confident that participants from this pool would respond quickly (in accordance with the time-sensitive nature of our research question). Fifty-six Cardiff University undergraduates between the ages of 18 and 29 responded to our call and took part in Study 2 (mean age = 21.54 years, SD = 1.82; 47 female, 9 male). All participants provided informed consent and completed the study on a personal computer or mobile phone in exchange for £10 of remuneration. We conducted our data collection from March 18th − 28th, 2022, approximately four weeks after the February 2022 invasion of Ukraine.

### Materials and methods

We created 11 items intended to tap into the aspects of the three motivational factors identified in Study 1 that we expected to be most applicable to consumption of news surrounding the Russia-Ukraine War (Table 2). For example, “I read articles that keep me up-to-date on a current topic” (Study 1) was modified to “I consume news about the Ukraine Crisis to keep updated on current developments” (Study 2). Additionally, and although we had previously considered both negative and positive components of ‘Affect Regulation’ in Study 1, we only considered the negative components of ‘Affect Regulation’ in Study 2: due to the Russia-Ukraine War’s strong negative valence, we assumed that items tapping into positive valence would be less relevant to participants’ news-seeking behaviours. However, we do acknowledge that motivations to seek positive content could still be present: for example, readers might seek stories that highlight resilience or humanitarian efforts. Future research might explore the extent to which positive affect–related motivations influence news engagement during crises, and whether including such items improves construct comparability across studies In accordance with Niehoff et al.^13^ and with our preregistration, we also added two items designed to tap into ‘Self-Relevance’ motivations. All items were presented in random order.

**Table 2:** The 11 items used in study 2.

Questionnaire item	Motivational factor
I consume news about the Ukraine Crisis...	
To keep updated on current developments.	Informational updating
To get the latest information.	Informational updating
To formulate an opinion about the situation.	Informational updating
To understand the situation.	Understanding & sense-making
To give me a different perspective on the conflict.	Understanding & sense-making
Because it is my moral duty to stay informed.	Understanding & sense-making
Because it may be relevant to my personal situation.	Self-relevance
Because it may help me to prepare for an emergency situation.	Self-relevance
I *avoid* news about the Ukraine Crisis...	
Because it may distress me.	Affect regulation
Because it is difficult to watch.	Affect regulation
Because it may evoke emotions that I find difficult to handle.	Affect regulation

Participants responded to each item using a 6-point Likert-type scale (1 = “not at all applicable”; 6 = “strongly applicable”; note that we changed the scaling of this measure from Study 1 to Study 2 to make its range more consistent with the other measures used in Study 2; see https://osf.io/dwk7a/. for the full list of questionnaires). Additionally, participants rated how often they actively informed themselves about the Russia-Ukraine War using an 8-point Likert-type scale (1 = “never”; 2 = “less than once a week”; 3 = ”1–2 times a week”; 4 = “at least three times a week”; 5 = “once a day”; 6 = “2–3 times a day”; 7 = “at least four times a day”; 8 = “at least once per hour”; scale adapted from^50^ so that we could collect more fine-grained data surrounding participants’ news consumption habits than we previously had in Study 1). In practice, responses along this measure ranged from 2 (“less than once a week”) to 6 (“2–3 times a day”) (mean = 3.91, *SD* = 1.35), with only slight negative skew (= -0.06).

## Results

### Preregistered analyses

#### ‘Understanding & Sense-Making’ is the strongest predictor of news consumption surrounding the Russia-Ukraine war

In accordance with our pre-registered analysis plan, and after confirming low multicollinearity among the motivational factors (all VIFs < 2.83), we began by conducting a multiple regression to assess whether the three motivational factors previously identified in Study 1—‘Informational Updating’, ‘Understanding & Sense-Making’, and ‘Affect Regulation’—predicted how often participants actively informed themselves about the Russia-Ukraine War when the remaining factors were controlled. As in Study 1, we calculated participants’ scores along each factor by averaging their responses across all items corresponding to that factor (Table 2). We then excluded participants who were more than three standard deviations beyond the mean of any measure of interest (i.e., motivational factors: ‘Informational Updating’, ‘Understanding & Sense-Making’, ‘Affect Regulation’, ‘Self-Relevance’). Only one participant met these criteria, resulting in *N* = 55 for all further analyses. All factors were z-scored prior to analysis. In our regression model, only ‘Understanding and Sense-Making’ was a significant predictor of news consumption frequency, β = 0.40, 95% CI [0.05, 0.74], *t*(51) = 2.32, *p* = .024, indicating that participants with stronger motivations to understand the Russia-Ukraine War actively informed themselves about the war more frequently. No other factors were significant, all *p* values > 0.450.

Next, as pre-registered, we conducted a second multiple regression to assess whether all four motivational factors measured in Study 2—‘Informational Updating’, ‘Understanding & Sense-Making’, ‘Affect Regulation’, and ‘Self-Relevance’—predicted news consumption frequency (all VIFs < 2.84). Once again, ‘Understanding and Sense-Making’ was the sole significant predictor, β = 0.40, 95% CI [0.05, 0.75], *t*(50) = 2.27, *p* = .027; all other *p* values > 0.453 (Fig. 4B).

Notably, ‘Informational Updating’ was *not* an independent predictor of news consumption surrounding the Russia-Ukraine War in either analysis, even though it was the strongest predictor of *general* news consumption in Study 1. We revisit this discrepancy in the General Discussion.

Of course, because we used slightly different questionnaire items in Studies 1 and 2 (see Method), we also acknowledge that the altered phrasing and/or exclusion of specific items might have impacted our findings. Thus, in an exploratory analysis, we next sought to replicate the relationships between the individual motivational factors and news consumption frequency that we observed in Study 1.

### Exploratory analyses

#### ‘Informational updating’, ‘understanding & sense-making’, and ‘affect regulation’ individually predict specific news consumption frequency, but ‘self-relevance’ does not

**Fig. 5:**
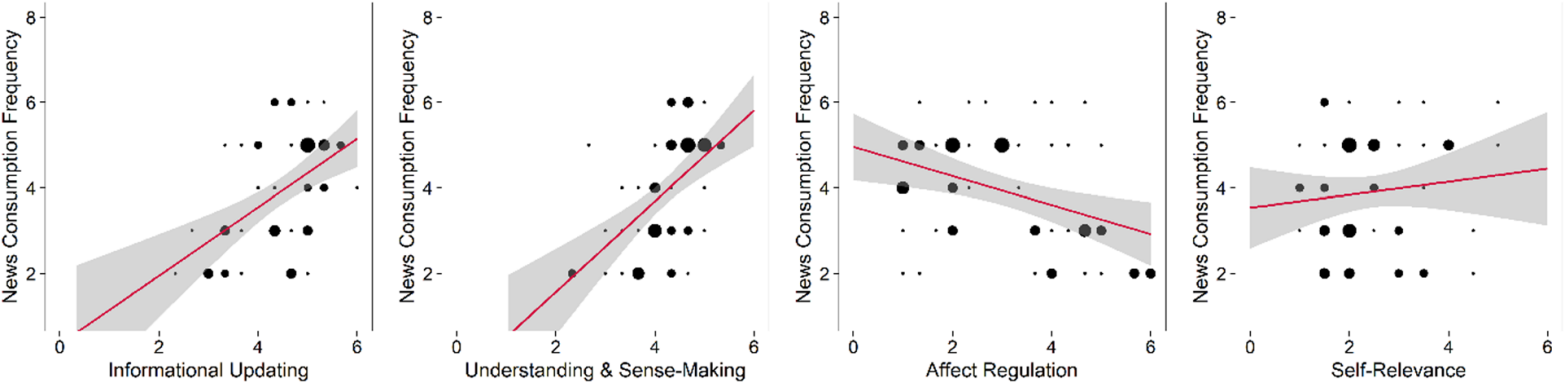
‘Informational Updating’, ‘Understanding & Sense-Making’, ‘Affect Regulation’, and ‘Self-Relevance’ as individual predictors of news consumption frequency in Study 2 (1 = “never”; 8 = “at least once per hour”). The points correspond to real data, with the size of the points reflecting the number of observations at each level.

As for the analyses in Study 1, we next assessed whether the four motivational factors considered in Study 2—‘Informational Updating’, ‘Understanding & Sense-Making’, ‘Affect Regulation’, and ‘Self-Relevance’—were individually predictive of news consumption frequency. To do so, we conducted four separate linear regressions with Bonferroni corrections to account for multiple comparisons (Fig. 5).

As in Study 1, both ‘Informational Updating’ and ‘Understanding & Sense-Making’ were positively predictive of news consumption frequency (‘Informational Updating’: *b* = 0.80, 95% CI [0.43, 1.18], *t*(53) = 4.28, adjusted *p* < .001; ‘Understanding & Sense-Making’: *b* = 1.06, 95% CI [0.63, 1.50], *t*(53) = 4.87, adjusted *p* < .001).

Conversely, ‘Self-Relevance’ was not predictive of news consumption frequency, *b* = 0.15, 95% CI [-0.21, 0.51], *t*(53) = 0.85, adjusted *p* = .399. Given that ‘Self-Relevance’ previously predicted consumption of COVID-19-related news^13^, this finding was somewhat unexpected. We revisit this point in the General Discussion.

Finally, and contrary to Study 1, ‘Affect Regulation’ was negatively predictive of news consumption frequency, *b* = -0.34, 95% CI [-0.57, -0.11], *t*(53) = -3.00, adjusted *p* = .016. We interpret this finding with caution, given that ‘Affect Regulation’ was nonsignificant when controlling for the remaining motivational factors (see above). Nevertheless, and relative to general news consumption (Study 1), the strong negative valence of the Russia-Ukraine War could reduce news consumption among participants who are more strongly motivated to maintain positive affect.

### ‘Affect regulation’ May be nonlinearly related to news consumption surrounding the Russia-Ukraine war

Interestingly, Niehoff et al.^13^ observed a quadratic relationship between headline choice and anticipated affect. In the context of COVID-19-related news, participants were more likely to choose articles that they expected to induce moderate levels of negative affect, relative to articles that they expected to induce either strong negative affect or no affective response. In an exploratory analysis, we asked whether the relationship between ‘Affect Regulation’ and news consumption surrounding the Russia-Ukraine War might likewise be nonlinear, such that participants with moderate motivations to avoid negative affect (i.e., moderate scores along ‘Affect Regulation’) might consume news most frequently.

**Fig. 6:**
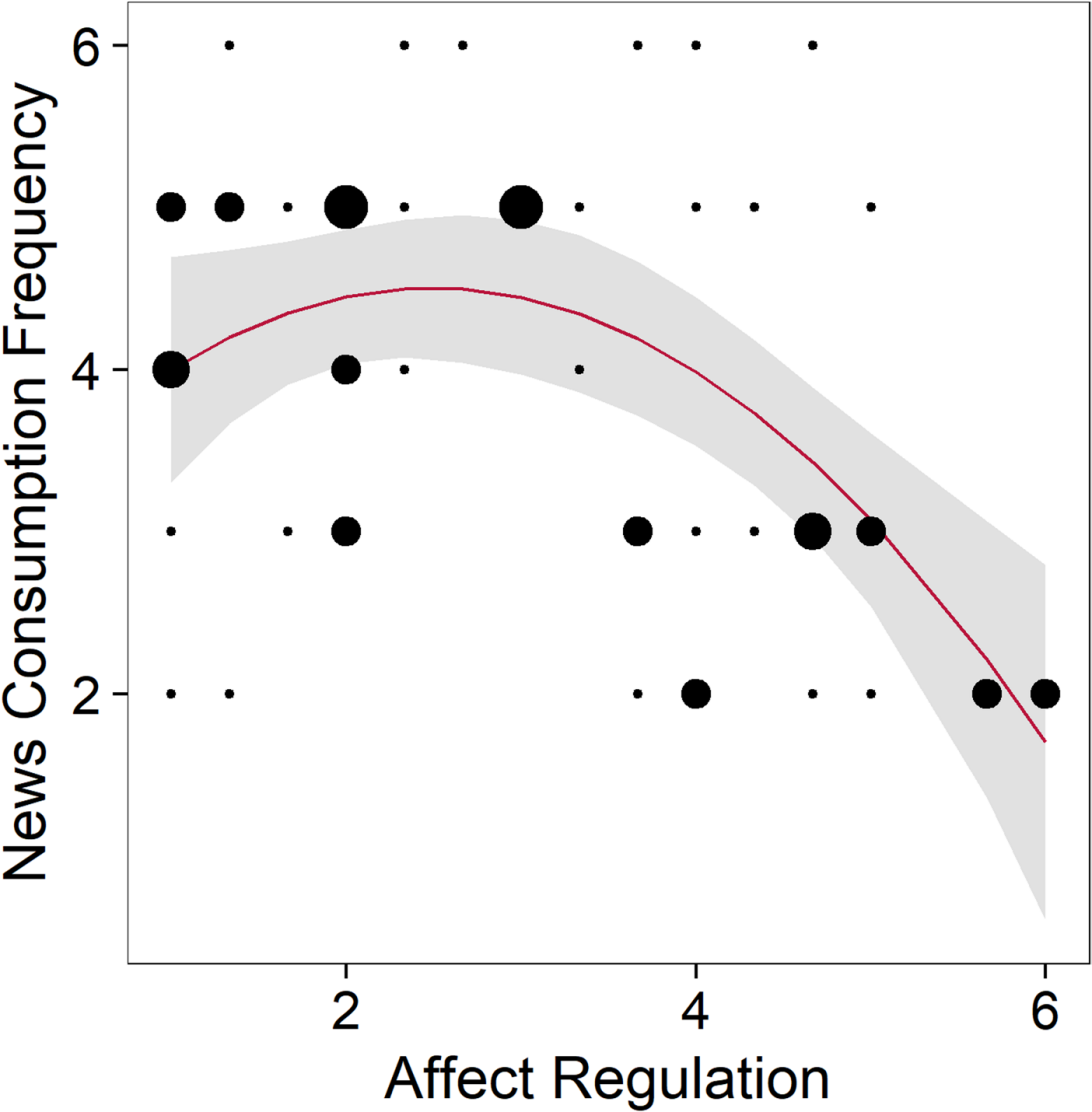
The quadratic relationship between ‘Affect Regulation’ and news consumption frequency observed in Study 2. The points correspond to real data, with the size of the points reflecting the number of observations at each level. The red line corresponds to model-predicted values, and the grey band reflects to the 95% confidence interval.

When ‘Affect Regulation’ was considered as an individual predictor of news consumption frequency (i.e., separately from the remaining three motivational factors), a quadratic model that included both a linear term (‘Affect Regulation’) and a quadratic term (‘Affect Regulation’^2^) provided a significantly better fit to the data than a simple linear model, *F*(1, 52) = 8.36, *p* = .006 (linear model R^2^ = 0.15; quadratic model R^2^ = 0.26). The quadratic term was significantly and negatively predictive of news consumption frequency, *b* = -0.23, 95% CI [-0.39, -0.07], *t*(52) = -2.89, *p* = .006. Relative to this quadratic model, introducing an additional cubic term (‘Affect Regulation’^3^) did not significantly improve model fit, *F*(1, 51) = 0.45, *p* = 0.505 (quadratic model R^2^ = 0.26, cubic model R^2^ = 0.27). Thus, and in agreement with Niehoff et al.^13^, participants with moderate motivations for ‘Affect Regulation’ consumed news surrounding the Russia-Ukraine War more frequently than participants with strong or weak motivations for ‘Affect Regulation’ (Fig. 6).

As this analysis was exploratory, this finding should be interpreted with caution. Nevertheless, it can readily be accommodated by existing theoretical frameworks. For example, studies examining the effect of arousal on performance have found that too little arousal impedes motivation, whereas too much arousal induces stress and anxiety; thus, optimal performance is best supported by moderate arousal^53^. Further exploration of these ideas as applied to news consumption may prove a promising direction for future research.

## General discussion

Each day, we are exposed to more news than we can possibly consume. Although the factors that lead us to select specific news items are of interest to news providers and researchers alike, they remain poorly understood. In Study 1, we identified three factors that promote general news consumption: ‘Informational Updating’, ‘Understanding & Sense-Making’, and ‘Affect Regulation’.

These three motivational factors are closely connected to Uses and Gratifications Theory^3–5^, which posits that consumers actively seek out media in effort to fulfill specific needs (including the need to stay informed and the need to reduce stress, among others). However, our approach differs from Uses and Gratifications Theory in that we took a bottom-up, data-driven approach towards determining which factors might drive consumption habits, rather than defining those factors a priori (see^52^. Additionally, we extend this approach in effort to determine which factors are most influential in facilitating general news consumption (Study 1) as well as in facilitating news consumption surrounding specific topics (Study 2).

In our sample, general news consumption was predominantly driven by motivations for ‘Informational Updating’. This finding is consistent with existing media strategies^6^ and theoretical perspectives^10,14^. We also expanded this prior work by demonstrating that the influence of ‘Informational Updating’ extends beyond the influence of trait curiosity, a personality measure known to promote real-world information seeking^34,54^. These data suggest that ‘Informational Updating’ is not simply a manifestation of trait curiosity, but rather an (at least partially) distinct motivational factor that facilitates news consumption.

Nevertheless, and despite the fact that ‘Informational Updating’ motivated general news consumption in our sample, it may not always motivate specific news consumption. In Study 2, we found that consumption of news surrounding the Russia-Ukraine War was predominantly driven not by ‘Informational Updating’, but instead by ‘Understanding & Sense-Making’.

There are several reasons why the motivational factors underlying general and specific news consumption might differ. Of course, the specific nature of the Russia-Ukraine War might encourage particular motivational factors to take precedence. For example, the complex socio-political context surrounding the war could lead readers to feel that, although they may generally know *what* is happening (e.g., through incidental news exposure;^54–58^, they may not understand *why*. Such perceptions might increase the motivation for ‘Understanding & Sense-Making’^10^. Notably, the Russia-Ukraine War also escalated at a time marked by increasing levels of news fatigue and avoidance^1^, which might have also decreased motivations for ‘Informational Updating’ relative to what we observed in Study 1.

An alternative possibility is that, relative to general questionnaires, questionnaires concerning specific news topics might better index participants’ actual consumption habits. Specific questionnaires provide focused, tangible points of reference that might lead to more accurate reporting of real-world behaviour^59^. Future research could explore this possibility by targeting headlines towards particular motivational factors, then investigating whether participants’ self-reported motivations map onto the headlines that they choose to consume (see^13^ for a similar approach). Importantly, different motivations might also facilitate different forms of news consumption. For example, people who are strongly motivated by ‘Informational Updating’ might spend more time scrolling through headlines, but less time reading full articles relative to people who are strongly motivated by ‘Understanding & Sense-Making’.

Additionally, it is important to acknowledge that the questionnaire items used in Study 2 differed slightly from the questionnaire items used in Study 1. In Study 2, our goal was to measure engagement with news that was specific to the Russia-Ukraine War; to this end, we adapted and refined several questionnaire items in effort to better reflect the specific context (for example we omitted the items related to positive affect under the assumption that these items might be less relevant in the context of a negatively-valenced event).

Importantly, and despite these modifications, we found that ‘Informational Updating’ and ‘Understanding & Sense-Making’ were significant individual predictors of news consumption in both Studies 1 and 2, suggesting consistency across samples and measurements. Nevertheless, our measurement changes also introduce variation that could at least partially account for the differences in factor structures observed across studies. This remains an important area of inquiry for future research.

Although we cannot conclusively determine why motivations to consume general and specific news might differ, the fact that they do differ has important implications for future research. As in Study 1, many studies investigating motivations for or attitudes towards news consumption employ general questionnaires^10,55,56^. Our findings suggest that future research may benefit from a nuanced approach that takes into account that dominant motivational factors might vary across different news topics.

Of course, we also acknowledge that the three motivational factors identified in Study 1—and the prominent influences of ‘Informational Updating’ and ‘Understanding & Sense-Making’ identified in Studies 1 and 2, respectively—may be sample-dependent. We opted to sample from an undergraduate population because, relative to older audiences, young adults consume less news via traditional outlets^51^ and are less likely to actively seek out news content, instead gaining much of their information through incidental exposure^55–57^. The motivational factors that drive young adults to seek these outlets despite their higher rates of disengagement are thus of strong theoretical interest, because news-seeking can help readers become informed and empowered participants in their local and global communities^60^.

Nevertheless, the factors that supported news consumption among our samples in Studies 1 and 2—both comprising young adult, UK-based, predominantly female undergraduates—do not necessarily extend to the general population. In particular, we note that in Study 1, we did *not* find any evidence that ‘connectedness’ motivates news consumption. This finding contrasts with prior research, which supports that news can serve a clear social function^10^. However, these considerations may not apply to the particular news contexts considered in the present study and/or to the way that young adults interact with traditional news outlets, which were the focus of our questionnaire items .

Similarly, in Study 2, we found no evidence that ‘Self-Relevance’ impacted consumption of news surrounding the Russia-Ukraine War, even though it does predict COVID-19 related news consumption^13^. This finding may reflect the fact that most participants within our UK-based sample did not perceive the Russia-Ukraine War as being particularly self-relevant, perhaps due to its relative geographical remoteness.

In summary, our data contribute to a strengthened theoretical understanding of why young adults consume news, both in general (Study 1) and with regard to specific news topics (Study 2). In addition to informing theoretical perspectives surrounding information seeking and media psychology, our data also highlight several promising directions for collaborative research between experimental psychologists, media researchers, and news providers. For example, future research might explore (1) which motivational factors underscore the consumption of other specific news topics, as well as (2) how the motivational factors identified in the present study relate to subjective experiences of news consumption, including factors such as information overload and news fatigue. In turn, these findings could help tailor news content such that it promotes regular engagement while also facilitating healthy news hygiene. We look forward to future research examining how and why individuals differ in their self-reported motivations to consume news, as well as how these differences manifest in real-world news-seeking behaviour.

## Data Availability

The datasets generated and/or analysed during the current study are available in the Open Science Framework (OSF) repository, https://osf.io/dwk7a/.
